# Concentrated growth factors: basic characteristics and clinical application

**DOI:** 10.1515/med-2026-1508

**Published:** 2026-08-20

**Authors:** Voja Pavlovic, Jelena Vucic, Milena Trandafilovic

**Affiliations:** Department of Physiology, Medical Faculty University of Nis, Nis, Serbia; Department of Neonatology, Children’s Hospital, University Clinical Centre Nis, Nis, Serbia; Department of Anatomy, Medical Faculty University of Nis, Nis, Serbia

**Keywords:** concentrated growth factors, platelets, growth factors, platelet concentrates

## Abstract

**Introduction:**

The beneficial activity of platelet concentrates (PC), during healing process is mainly attributed to the regenerative potential of growth factors (GF) released from platelets. Since concentrated growth factor (CGF) possesses great regenerative properties, in recent years has gained increased attention for regenerative procedures.

**Content:**

Based on a comprehensive analysis of recent basic and clinical studies on CGF, present review summarizes the most used procedures for CGF, PRF (platelet rich fibrin) and PRP (platelet rich plasma) preparation and simultaneously clarify advantages and disadvantages of regenerative properties among CGF, PRF and PRP. Also, current review intends to clarify the physiologic role of certain CGF components and to analyze their potential clinical applications in regenerative medicine.

**Summary and Outlook:**

This form of PC is completely autologous product, requires one centrifugation step with alternating speed for production and easy to prepare with minimal expenses. Furthermore, CGF contains high amount of GF, which are continuously released for days, and formed fibrin matrix is more stable compared to the other forms of PC (PRP and PRF). Furthermore, some limitations of CGF application and points which should be resolved by incoming research studies to further elucidate the CGF protocol standardization and potential effect of leukocytes.

## Introduction

Different autologous platelets concentrates (APCs), have been widely utilized in regenerative medicine due to its ability to enhance the healing process and tissue regeneration. Platelets enclose more than 1,500 diverse bioactive molecules, including most of all growth factors, cytokines, chemokines and others [1]. All these molecules among other roles, have been shown to have key role on cell proliferation, differentiation, migration and matrix remodeling, which are responsible for accelerated tissue repair and improved regenerative benefits [2]. By using autologous growth factors, derived from the patient’s own peripheral blood, additional progress in tissue healing may be accomplished in various regenerative procedures. Furthermore, autologous nature of platelet concentrates diminish possibility of disease transmission and immune reaction [3]. Even there are different procedures for APCs preparation (different centrifugation steps, with or without of anticoagulant solution or some activator of platelets), these concentrates finally result with formed fibrin scaffolds enriched with bioactive molecules which may regulate various cell actions [4].

Based on their characteristics and methods used for their preparation, the main generation of APCs are platelet-rich plasma (PRP), platelet-rich fibrin (PRF) and concentrated growth factors (CGF). PRP, as the first generation of APCs, have been used widely in different clinical fields [5]. However, there are various protocols for PRP preparation and lack of standardization method makes complicated to compare potential benefits of this procedure. Also, PRP preparation requires the addition of anticoagulants which may interfere with natural healing process and thrombin or calcium chloride (for fibrin polymerization induction) which are able to rise concerns about its biosafety [5], 6]. To fulfill the stated drawbacks of PRP, the second generation of APCs (PRF) overcomes the need for exogenous thrombin or application of any anticoagulant agents, with only one centrifugation step. PRF production protocol enables formation of dense fibrin network with resulting delayed release of growth factors during wound healing [7]. Previous studies confirm these observations, showing the continual and steady release of growth factors up to 10 days [8], 9]. Even modification of centrifugation speed and time provided different forms of PRF, as we reviewed recently [2], there are few standardized PFR protocols with reduced expense and simple method for production compared to the PRP [10].

By using single centrifugal force, the species of growth factors in PRF are relatively simple [1] and with relatively low content [11]. Therefore, third generation of APCs (CGF) is provided to overcome observed limitations, by centrifuging blood samples at alternating and controlled speed [12]. The process of different centrifuge speeds allows formation of a denser fibrin matrix which is more stable and contains more growth factor and cells compared to the PRP and PRF [3], 6]. Growth factors are trapped in the fibrin matrix and they are released slowly, imitating the natural process of tissue healing [13]. Simultaneously, gradually releasing of growth factors reduces possibility of potential inflammatory response which may occur due to excessive concentrations of growth factors [14].

Therefore, the current review intends to summarize the preparation procedures and regenerative properties of PRP, PRF and CGF, as well as to provide basic physiologic characteristics of certain CGF components in tissue regeneration and to evaluate potential clinical application of CGF and some limitations which should be resolved by forthcoming research.

## Methodology

We conducted a comprehensive search to identify relevant studies investigating physiological role of concentrated growth factors and their possible clinical application. Electronic databases, including PubMed and Web of science, were searched from their inception to October 31, 2025. The search strategy used terms like “concentrated growth factors,” “physiological role” and “clinical application.” Initially, collected studies were analyzed according to their titles and abstracts to identify their potential relevance to the research topic. Eligible articles were published in English, original research involving human and animal studies, systematic reviews or meta-analyses. Studies were excluded if they are not peer-reviewed, including conference abstracts, editorials and commentaries. The eligibility of the studies through selection process was estimated by two reviewers (JV and MT) while if any uncertainty occurs, a third reviewer (VP) was asked to reach consensus.

### Preparation of APCs

Recently, regenerative medicine has focused on autologous growth factors, which bind to specific cells and guide them towards migration, differentiation, proliferation and cell secretion [15]. The large amount of growth factors stored in platelets, including PDGF (platelet-derived growth factor), TGF-β1 (transforming growth factor β), VEGF (vascular endothelial growth factor) and IGF-1 (insulin growth factor-1), are involved in angiogenesis, matrix remodeling and tissue regeneration [16]. The efficacy of APCs in tissue regeneration mainly depends on the amount of the secreted growth factors from platelets. Therefore, appreciable scientific interest has been shown in developing methods and protocols to obtain adequate amount of growth factors from APCs.

### Platelet-rich plasma (PRP)

**Figure 1:**
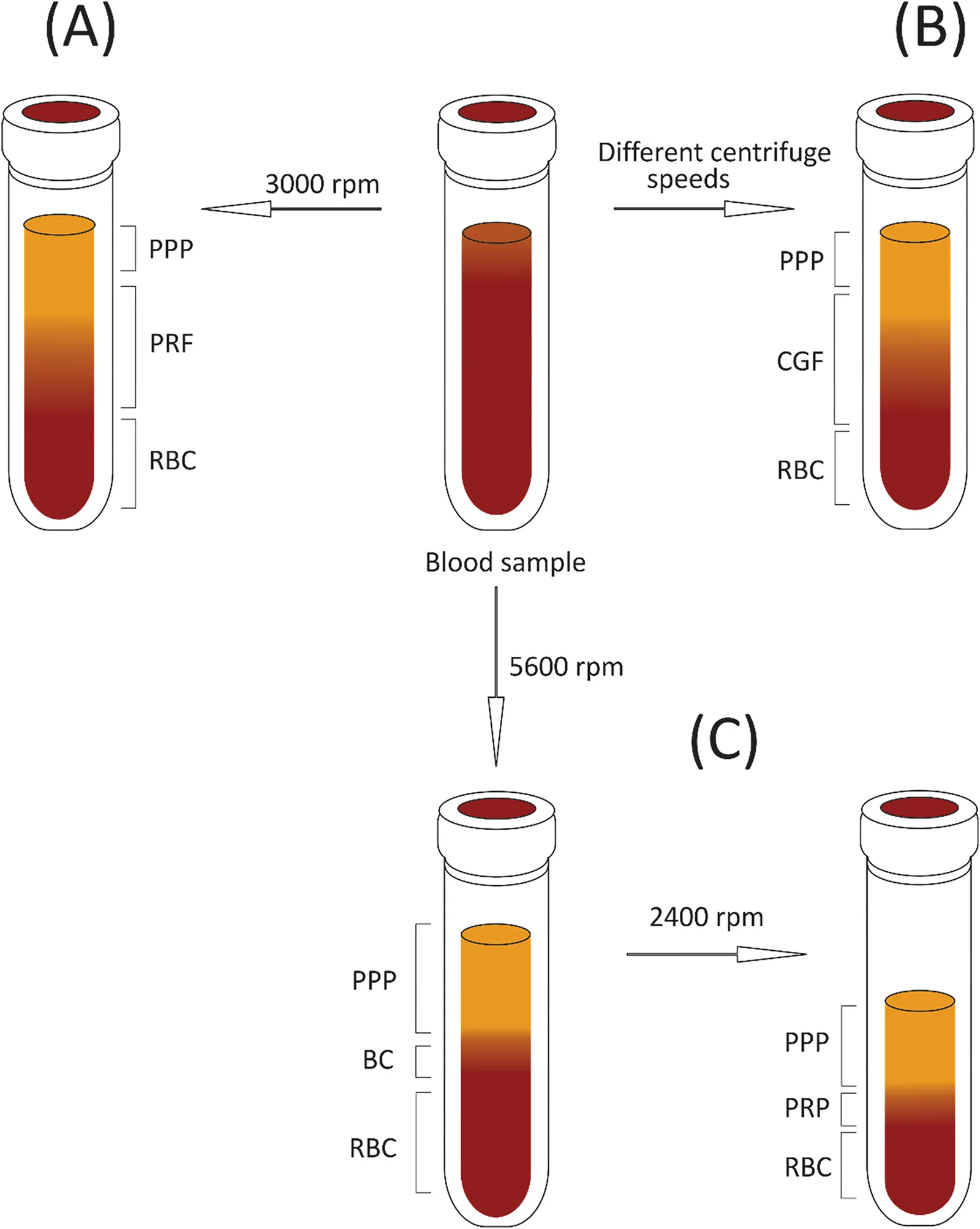
PRP, PRF and CGF production procedure. (A) PRF production procedure. Venous blood is collected without any anticoagulants and centrifuged 10 min at 3,000 rpm. Following the centrifugation process, three layers are formed: Bottom layer-RBC, top layer PPP and middle layer-containing fibrin clot, termed as PRF. (B) CGF production procedure. Blood sample is centrifuged without any anticoagulant at alternating speeds (2 min at 2,700 rpm, 4 min at 2,400 rpm, 4 min at 2,700 rpm and 3 min at 3,000 rpm). This procedure ends with formation of three layers: Bottom layer-RBC, top layer-PPP and middle layer-CGF. (C) PRP production procedure. Venous blood is collected with anticoagulant and centrifuged at 5,600 rpm which provides formation of three layers: PPP (top), BC (middle) and RBC (bottom). BC and PPP are collected and transferred to sterile tube, but without anticoagulant solution, and proceeded for next centrifugation step at 2,400 rpm. Second centrifugation step results with formation of PRP (middle layer). Separated PRP is collected and activated by adding a mixture of thrombin and calcium chloride. Abbreviations: PRP-platelet rich plasma, PRF- platelet rich fibrin, CGF-concentrated growth factors, RBC-red blood cells, PPP-platelet poor plasma, BC-buffy coat.

PRP represents the first generation of APCs. During preparation of PRP, it is necessary to use anticoagulant solution (mainly citrate-phosphate-dextrose solution) and nonautologous thrombin (bovine thrombin and calcium chloride, for platelet activation). Even today there are 40 different commercial systems able to create PRP from whole blood [5], two centrifugation steps are essential for PRP production (separation and concentration). Namely, the whole blood (8 mL) is collected in a sterile tube containing citrate-phosphate-dextrose solution and centrifuged at 5,600 rpm [12], 17]. Following the first centrifugation, cell are separated in three layers: top layer contains platelet-poor plasma (PPP), middle layer is called buffy coat (BC) which contains platelets and white blood cells and bottom layer which contains red blood cells. BC and PPP are collected and transferred to sterile tube, but without anticoagulant solution and proceeded for next centrifugation step at 2,400 rpm. Second centrifugation step results with formation of PRP (middle layer), which is located between top layer (PPP) and bottom layer (red blood cells). Separated PRP is collected and activated by adding a mixture of thrombin and calcium chloride. Within 30 s fibrin mesh is formed and platelet degranulation occurs with resulting releasing of growth factors (Figure 1). Only then fibrin gel is ready to use and placed to the desirable site [3], 12]. Also, it is necessary to point out that multiple protocols used to prepare PRP resulted in formation different PRP categories. Based on the total platelet and leukocyte content, two different PRP categories have been proposed (leukocyte-rich PRP and leukocyte reduced PRP), with modified biological characteristics, as we reviewed earlier [5].

### Platelet-rich fibrin (PRF)

Second generation of APCs, as a new alternative to PRP, was introduced in France, back in 2001, by Chouckroun et al., as we discussed earlier [2]. This APC does not require platelet activator (thrombin or calcium chloride) or any anticoagulant solution. Therefore, without any anticoagulant, clot formation occurs naturally and there is no risk associated with the use of bovine thrombin [5], 6]. Only centrifuged autologous blood is needed for PRF production. Briefly, venous blood is collected in 10 mL sterile glass tubes, without any anticoagulant solution, and centrifuged 10 min at 3,000 rpm. Centrifugation step results with formation of three layers: top layer (PPP), middle layer (fibrin clot, known as PRF) and bottom layer (red blood cells). PRF clot is effortlessly collected by discarding the upper layer (PPP). The obtained PRF is usually called leukocyte PRF of Choukroun’s PRF (Figure 1) [18]. Since there is no use of any anticoagulant, the coagulation process starts immediately, platelets are activated upon contact with glass tube with resulting release of clotting factors [17]. The essential value of PRF production is to permit the platelet activation and fibrin polymerization in a physiological manner. Analysis of formed PRF clot reveals hugely entraps growth factors, platelets, macrophages, granulocytes and neutrophils [19]. Platelets have crucial role in initial phase of healing while leukocytes, beside their role in anti-inflammatory and antibacterial response, may also promote the wound healing process [20]. Similar to PRP, modification of basic PRF protocol provided various types of PRF. Changing the speed and duration of centrifugation introduced modified types of PRF, including A-PRF (advanced PRF), A-PRF + (advanced PRF plus), i-PRF (injectable PRF), T-PRF (titanium PRF), with different biological activity and potential uses, as we reviewed recently [2].

### Concentrated growth factor (CGF)

The third and final generation of APCs, termed as CGF, was introduced back in 2006 by Sacco, as was previously reported [21]. Similar to the PRF, addition of anticoagulant solution or some activator of platelets is not necessary for CGF production. This procedure requires only one centrifugation step, by using special centrifuge (Medifuge MF200, Silfradent srl, Italy), with alternating speeds. Namely, whole blood sample is collected in glass-coated plastic tubes, without any anticoagulant solution, and centrifuged in following manner for 13 min: 2 min at 2,700 rpm, 4 min at 2,400 rpm, 4 min at 2,700 rpm and 3 min at 3,000 rpm. Following the centrifugation step, three layers are formed: top layer (PPP), middle layer (CGF) which contains growth factors and fibrin and bottom layer (red blood cells). Middle layer of CGF is easily removed by using surgical scissors and CGF (solid form) is then placed over the target site (Figure 1) [14], 22].

**Figure 2:**
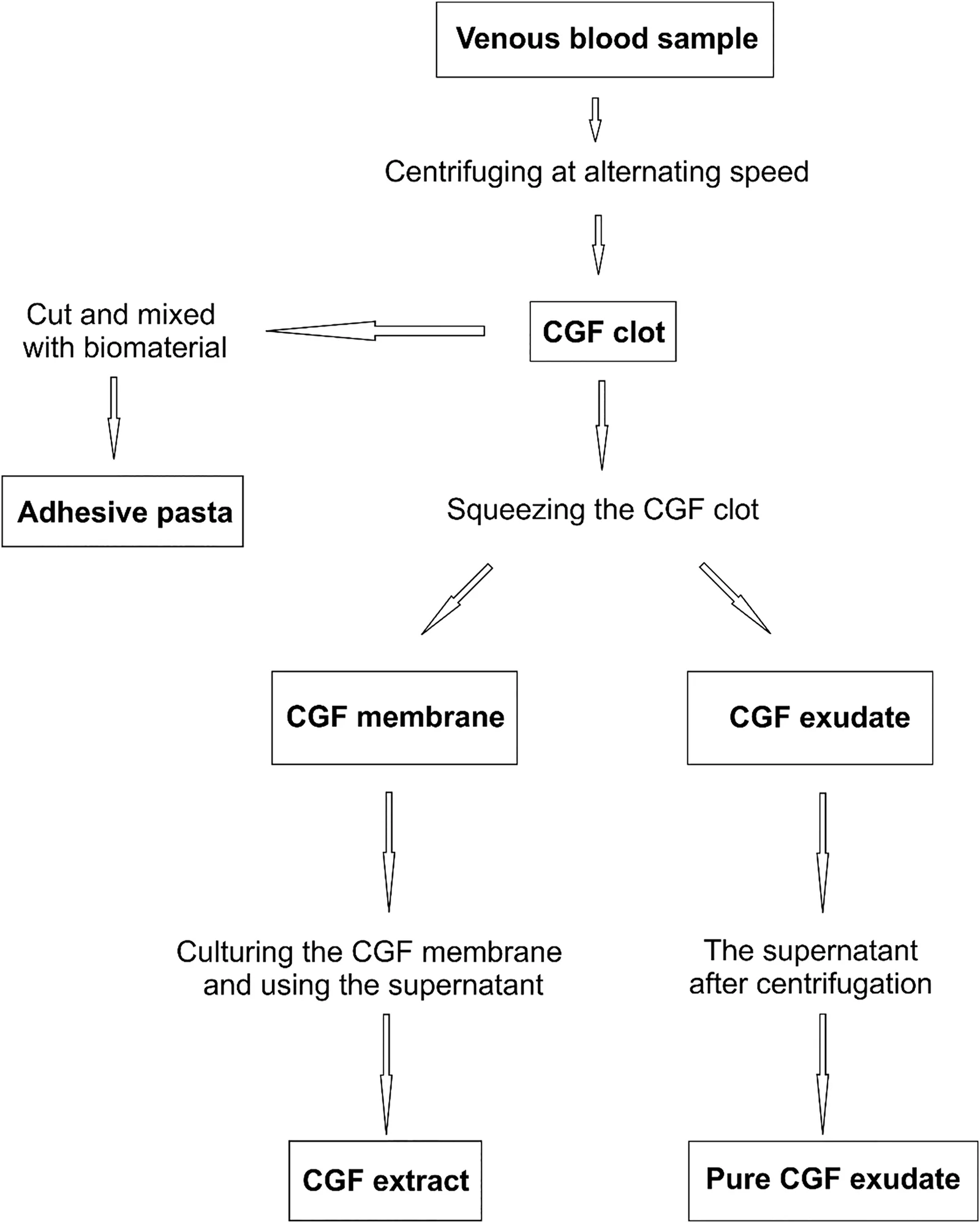
Production steps for different types of concentrated growth factor (CGF) preparation. Figure illustrates principal mechanism for production various CGF types. By squeezing the CGF clot, released liquid may be used for preparation of CGF exudate or pure CGF exudate. Simultaneously, obtained CGF membrane may serve for CGF extract production (after cultivation period of 7–14 days). Furthermore, acquired CGF clot may be useful for adhesive pasta preparation (by mixing with certain biomaterials).

CGF membrane is produced from CGF in solid form by squeezing (1–2 min) the obtained CGF clot in a special box, until it reaches the thickness of 1 mm [23]. This CGF membrane releases growth factor slower than CGF in solid form [21] and may be used immediately or for further production of CGF extract. CGF extract is usually produced by placing the CGF membrane in culture medium for couple of days (7–14 days). After cultivation period is ended, culture medium is collected and centrifuged. Obtained supernatant (CGF extract) may be used in further research, after it is enriched with fetal bovine serum and antibiotics [24]. On the other hand, released liquid from CGF clot squeezing, during CGF membrane preparation, represents another form of CGF, termed CGF exudate. Centrifugation of the obtain fluid (5 min at 3,000 rpm) leads to removal of red blood cells and collected supernatant is pure CGF exudate [25]. In addition, CGF clot may be cut into small pieces and mixed with biomaterials and further placed, as an adhesive paste, in target place (Figure 2) [6].

### Bioactive factors in CGF and clinical application

Growth factors represent key factors in regeneration mechanism and have essential role in regulating processes involved in tissue remodeling and regeneration [12]. However, since the different protocols are used for production the numerous types of APCs, obtain APCs show inequality in total amount of growth factors and kinetics of growth factor release. Accordingly, amount of growth factors and their release kinetics may have key role in determining the efficiency of CGFs and other APCs. The ability of CGF production protocol to use only one centrifugation step at alternating speeds, allows formation of dense fibrin network accommodating platelets, leukocytes, CD34 + cells and growth factors (Table 1) [16]. Furthermore, growth factors and cells are more abundant in CGFs compared to the PRP and PRF [3], 6]. Entrapped growth factors are released steadily over time [26], enabling controlled and continued regenerative process and diminish inflammatory response development due to huge growth factor concentrations [14]. In line with previous findings, it has been shown that arrangement of fibrin network and concentration of leukocytes may have impact on the growth factors release from APCs [27], indicating possible factors included in effectiveness of CGFs (Table 2).

**Table 1:** Certain growth factors in CGF and their basic physiologic function.

Name (abbreviation)	Function
Vascular endothelial growth factor, VEGF	Involved in process of angiogenesis; stimulates endothelial cell proliferation, migration and survival; enhances permeability of the vessels; induces chemotaxis of leukocytes
Platelet derived growth factor, PDGF	Provides migration, proliferation and survival of mesenchimatous cell lineage; stimulates angiogenesis and TGF- β secretion from macrophages; enhances collagen synthesis and proliferation of bone cells
Transforming growth factor β (TGF- β)	The most abundant growth factor in CGFs; stimulates collagen synthesis and prevents its breakdown; inhibits bone resorption and osteoclast formation; regulates cell proliferation, differentiation and tissue remodeling; promotes angigenesis
Epidermal growth factor, EGF	Promotes angiogenesis and epithelization; induces cytokines secretion from epithelial and mesenchymal cells; shortens the healing time
Insulin growth factor-1, IGF-1	Provokes differentiation and mitogenesis of mesenchymal cells; stimulates collagen synthesis, chemotaxis and activation of osteoblasts; induces antiapoptotic effects and enhances cell survival
Bone morphogenetic protein 2, BMP-2	Enhances osteoblast differentiation and maturation; provide key role in bone and cartilage development; stimulates regenerative process

**Table 2:** Key differences of PRP, PRF and CGF in preparation protocol, growth factor release, clinical application and limitations.

	PRP	PRF	CGF
Preparation protocol	Lack of standard preparation protocol; More than 40 different preparation protocols	More standardized preparation protocol; Modification of standard preparation protocol provides various types of PRF (A-PRF, A-PRF +, i-PRF and T-PRF)	More standardized preparation protocol; provides CGF in solid form; Modification of CGF in solid form results with formation of CGF membrane, CGF extract, CGF exudate, pure CGF exudate
Growth factor release	Rapid delivery of growth factors; Growth factor are released within the first hour of application	Steady release of growth factors up to 10 days; Some type of PRF (A-PRF) releases of certain growth factor up to 14 days	Steady release of growth factors up to 14 days; Certain growth factors may be released up to 28 days
Clinical application	Wound healing Augmentation of maxillary sinus Handling of periodontal intrabony defects Enhances graft placement and stability	Wound healing Coverage of gingival recession Treatment of intrabony defects Procedure of sinus augmentation	Wound healing Treatment of periodontal defects Coverage of gingival recession Osteoinductive potential of CGF Stimulates osteogenic differentiation of bone marrow stem cells
Advantages	Worldwide application; Delivery a huge amount of growth factors in short time period	Longer releasing of growth factors vs. PRP; imitating nature healing process; Preparation requires no anticoagulants or thrombin activators; Easy to prepare and requires only one centrifugation step	Steady and long growth factor releasing; Preparation requires no anticoagulants or thrombin activators; Preparation needs only one centrifugation step and it is inexpensive
Limitations	Without standard protocol for PRP preparation; Not completely autologous product; Using of anticoagulants and fibrin activators may alter healing process and induces coagulopathies	Since is used without anticoagulants, it requires preparation quickly completed; Not large quantities can be produced at once	Used blood volume and time for CGF preparation may influence the final composition of CGF; Alterations in blood pH may influence the platelet count in CGF and final CGF composition

Released growth factors bind to the cellular transmembrane receptors and regulate different intracellular signaling pathways [28]. However, obtained growth factors from APCs are released in biologically relevant ratio, enabling the concept of using a balanced set of bioactive growth factors, rather than using high amounts of exogenous growth factors [29]. Also, the kinetics of growth factors release affects their bioactivity. In line with this, previous findings showed that growth factors release in PRP occurs within 1 h of application, indicating their role only in the first phase of tissue regeneration [30]. On the other hand, growth factors releasing in PRF takes place up to 10 days, while in some types of PRF (A-PRF) it has been shown, in *vitro* conditions, to last up to 14 days [30]. It has been reported that CGF contains more amount of growth factors compared to the other APCs and different kinetics of releasing [22], implying the ability of CGF to support different phases of regeneration process. Earlier reports showed growth factor release from CGFs up to 13 days [31] and 28 days [14]. It should be noted that these results were obtained from *in vitro* studies, by evaluating the growth factors release from CGF extract. To further prove CFG effectiveness and safety for clinical application, additional and larger clinical studies are necessary. Furthermore, these findings indicate gradually releasing of VEGF up to 14 days when it reaches the peak, while BMP-2 (bone morphogenetic protein-2) and TGF-β1 reach peak at 21 days and then gradually decrease up to 28 days. Obtained results suggested that CGFs have ability for continuous release of different growth factors over time, with important role in complex process of tissue regeneration. On the other hand, such preclinical findings should be evaluated and confirmed by high-quality clinical studies. Therefore, according to the kinetics of growth factor release from CGFs, two different phases have been disclosed, including early and late phase [32]. During early phase growth factor are released mainly by activated platelets due to centrifugation process application and late phase where releasing of growth factors is result of fibrin structure degradation or new synthesis of growth factors by cells entrapped in CGFs. The late phase usually reaches peak at 14 days [30], 33].

Since various reports [13], 16] have demonstrated that CGFs contain large amounts of growth factors (TGF-β1, VEGF, PDGF, IGF-1), different studies evaluated proliferation and differentiation capability of CGFs on stem cells. Majority of the studies (in mesenchymal pulp and ligament stem cells) showed positive proliferation effect of CGFs on different stem cells in a dose-dependent manner [34], [35], [36]. On the other hand, some reports (on animal bone marrow stromal cells) demonstrated positive proliferation effect, in dose dependent manner, only when used low concentration CGFs were used [31], 37], while other studies (on human periodontal cells and on human stem cells from the apical papilla) stated no proliferation effect in a dose-dependent manner [21], 24]. Similar results were obtained when proliferation and differentiation capability of CGFs was evaluated in different adult cells or cell lines. Namely, most of these studies resulted with positive proliferation and differentiation capability of CGFs [38], 39], while some reported impaired cell proliferation rate [40] or positive proliferation effect with lower concentration of CGFs [41]. Improvement of cell proliferation and differentiation rate could be the result of a high concentration of growth factors in CGFs. Such discrepancy in obtained results may be results of different factors. Namely, since there is no standardized production protocol, finally produced CGF may contain various amounts of growth factors which makes it difficult to compare with others. Besides production protocol, total amount of blood used for CGF production, types of tubes used for CGF production and health status of donor used for CGF production, may influence the final CGF content and kinetics of growth factors release. All these factors, at the end, may influence on CGS regenerative potential. Moreover, most of the studies did not evaluate the total amount of growth factors after production which makes comparison more difficult, since it is well known that various number of platelet count in CGF may modify pH value of condition medium which leads to reduction of cell proliferation rate [42]. Also, majority of the reports were preclinical studies (including experiments *in vitro* with animal cells or studies with different cell lines) where findings are inadequate to compare with complex microenvironment *in vivo*, especially in humans. All these factors indicate that most desirable concentration of CGF vary among the studies and further research is needed to be optimized.

The presence of growth factors and fibrin scaffold, both with osteoinductive potential, initiated many studies to evaluate the role of CGF in the regeneration of bone defects. Different animal studies observed positive osteoinductive properties of CGF [38], 43], 44] while some reported no or limited effectiveness of CGF [45], 46] in regeneration of peri-implant bone defect and femur defect in animals. Furthermore, CGF has been reported to induce osteogenic differentiation of human bone marrow stem cells [47] and combination of CGF and stem cells shows higher regenerative potential than CGF alone [31], 37] in animal bone marrow stromal cells. Comparison of CGF with other APCs showed no significant difference in animal bone defect regeneration [46] while others reported higher effectiveness of CGF and A-PRF over the PRP in periosteal cell proliferation [22] and better regenerative potential of CGF than PRF in animal femoral defect repair [44]. Additionally, combination of CGF with grafts resulted with increased bone regenerative potential, in rat bone healing, than CGF alone [48]. Since the majority results of the osteoinductive potential of CGF comes from experimental studies, additional clinical trials are needed to confirm and to gain inside the regenerative process and safety application. Nevertheless, these inconsistent results may be due to presence of different amounts of growth factors and inflammatory cytokines which may result with inhibitory or stimulative effect on CGF [49]. Otherwise, osteoinductive properties of CGF may be attributed to two amino-acids (glutamic acid and taurine) present in CGF. Namely, taurine stimulates the osteoblasts differentiation [50] while glutamic acid may serve as a starting point for calcium phosphate mineralization [51]. Also, it should be noticed that obtained results come from a different studies (animal, *in vitro* or human studies) with distinct production protocols, which finally lead to CGF formation with heterogeneous growth factors amount. Lack of standardized CGF protocol may provide different kinetics of growth factors release and alter osteoinductive potential of CGF. Clear distinct should be also pointed out *in vitro* and *in vivo* reports, which may influence the final amount of growth factors in CGF and their later production induced by various cell signaling in more complex environment during *in vivo* studies. However, to resolve previous objectives, more prospective clinical trials are required.

Besides large content of growth factors, CGFs contain significant amount of matrix metalloproteinase (MMP), specific enzymes involved in degradation of all kinds of extracellular matrix proteins as well as in cell proliferation, differentiation and cell migration during tissue regeneration [12]. It has been demonstrated that CGFs release MMPs (MMP-2 and MMP-9) more promptly than growth factors with their peaks at 7 days [14], when CGF was cultured, without ant manipulation, for 28 days. Furthermore, higher amounts of MMPs are secreted in conditioned medium by CGFs compared to the starting levels of MMPs in CGFs, indicating the ability of entrapped cells in CGFs to produce and secrete MMPs. In line with these results, it has been demonstrated the presence of different cell populations, including monocytes, within CGFs [16]. It is necessary to point out that these findings were demonstarted by *in vitro* experiments and they still lack conformation from larger, case cotrolled, clinical trials. It is well documented that leukocytes have ability to produce many proteinases, including MMPs [5], 52]. Circulating monocytes could provide breakdown of extracellular matrix by releasing different MMPs (MMP-2, MMP-9 and MMP-13) [29], enabling elevated cellular migration through tissue with resulting increased efficiency of healing process [5]. Also, proteases have ability to control the activity of growth factors. Usually, TGF-β1 is stored bound to extracellular matrix. Secreted proteases degrade the matrix and induce the release of growth factor [5]. Released TGF-β1 is in inactive form and it is transform in active form by proteases [53]. Nevertheless, earlier findings showed that total number of leukocytes may influence the effectiveness of growth factors [27] primarily by extensive release of reactive oxygen species (ROS) in wounded tissue [54], 55]. Massive release of ROS occurs mainly to eliminate debris and potential microbes in damaged tissue with delayed healing process [56]. Therefore, total influx of leukocytes must be tightly regulated and further analysis might provide better understanding of potential role of leukocytes in regenerative process.

## Conclusions

CGF, as the newest formulation among APC generations with ability to constant deliver notable amount of growth factors to desirable location and enables angiogenesis with enhanced tissue regeneration, represents important therapy to address different injuries in regenerative medicine. This completely autologous material accommodate higher amounts of growth factors compared to the other APCs and requires low cost, simple and easy production. In addition, since present research showed no significant adverse effects, CGF provides safe and effective autologous material with fine immunological biocompatibility. However, current different results about the efficiency of CGF in regenerative process needs to be resolved. Majority of the obtained results come from experimental and preclinical studies. Furthermore, these findings need conformation from multiple controlled clinical trials to prove regenerative potential and safety of CGF treatment before it can be used as routine clinical treatment protocol.

On the other hand, lack of standardized CGF protocol makes difficult to compare obtained regenerative potentials of CGFs and to provide standardized treatment protocol. The optimal concentration of platelets, different concentrations of CFG among the donors, as well as different tubes and different blood volumes used for CGF preparation should be standardized and then evaluate the regenerative properties of CGF. Moreover, clarification of durable efficiency of CGF and its decomposition is also needed, especially in clinical applications. To address safety and potential clinical application in regenerative medicine, further randomized controlled trials and related researches are necessary, as well as meta-analyses and systematic reviews are also warranted to understand the additional benefits of CGF.

## References

[1] Yu M, Wang X, Liu Y, Qiao J (2019). Cytokine release kinetics of concentrated growth factors in different scaffolds. Clin Oral Invest.

[2] Pavlovic V, Ciric M, Jovanovic V, Trandafilovic M, Stojanovic P (2021). Platelet-rich fibrin: basics of biological actions and protocol modifications. Open Med.

[3] Mijiritsky E, Assaf HD, Peleg O, Shacham M, Cerroni L, Mangani L (2021). Use of PRP, PRF and CGF in periodontal regeneration and facial rejuvenation-A narrative review. Biology.

[4] Cakir S, Gultekin BA, Karabagli M, Yilmaz TE, Cakir E, Guzel EE (2019). Histological evaluation of the effects of growth factors in a fibrin network on bone regeneration. J Craniofac Surg.

[5] Pavlovic V, Ciric M, Jovanovic V, Stojanovic P (2016). Platelet rich plasma: a short overview of certain bioactive components. Open Med.

[6] Tabatabaei F, Aghamohammadi Z, Tayebi L (2020). In vitro and in vivo effects of concentrated growth factor on cells and tissues. J Biomed Mater Res.

[7] Shah R, Triveni MG, Thomas R, Mehta DS (2017). An update on the protocols and biologic actions of platelet rich fibrin in dentistry. Eur J Prosthodont Restor Dent.

[8] Kobayashi E, Flückiger L, Fujioka-Kobayashi M, Sawada K, Sculean A, Schaller B (2016). Comparative release of growth factors from PRP, PRF, and advanced-PRF. Clin Oral Invest.

[9] He L, Lin Y, Hu X, Zhang Y, Wu H (2009). A comparative study of platelet-rich fibrin (PRF) and platelet-rich plasma (PRP) on the effect of proliferation and differentiation of rat osteoblasts in vitro. Oral Surg Oral Med Oral Pathol Oral Radiol Endod.

[10] Kumar RV, Shubhashini N (2013). Platelet rich fibrin: a new paradigm in periodontal regeneration. Cell Tissue Bank.

[11] Li G, Wang H (2024). Novel applications of concentrated growth factors in facial rejuvenation and plastic surgery. Facial Plast Surg.

[12] Giannotti L, Di Chiara Stanca B, Spedicato F, Nitti P, Damiano F, Demitri C (2023). Progress in regenerative medicine: exploring autologous platelet concentrates and their clinical applications. Genes.

[13] Bonazza V, Hajistilly C, Patel D, Patel J, Woo R, Cocchi MA (2018). Growth factors release from concentrated growth factors: effect of β-tricalcium phosphate addition. J Craniofac Surg.

[14] Stanca E, Calabriso N, Giannotti L, Nitti P, Damiano F, Stanca BDC (2021). Analysis of CGF biomolecules, structure and cell population: characterization of the stemness features of CGF cells and osteogenic potential. Int J Mol Sci.

[15] Krafts KP (2010). Tissue repair: the hidden drama. Organogenesis.

[16] Rodella LF, Favero G, Boninsegna R, Buffoli B, Labanca M, Scarì G (2011). Growth factors, CD34 positive cells, and fibrin network analysis in concentrated growth factors fraction. Microsc Res Tech.

[17] Dohan DM, Choukroun J, Diss A, Dohan SL, Dohan AJ, Mouhyi J (2006). Platelet-rich fibrin (PRF): a second-generation platelet concentrate. Part I: technological concepts and evolution. Oral Surg Oral Med Oral Pathol Oral Radiol Endod.

[18] Dohan EDM, Rasmusson L, Albrektsson T (2009). Classification of platelet concentrates: from pure platelet-rich plasma (P-PRP) to leucocyte- and platelet-rich fibrin (L-PRF). Trends Biotechnol.

[19] Liu Y, Sun X, Yu J, Wang J, Zhai P, Chen S (2019). Platelet-rich fibrin as a bone graft material in oral and maxillofacial bone regeneration: classification and summary for better application. Biomed Res Int.

[20] Barbon S, Stocco E, Macchi V, Contran M, Grandi F, Borean A (2019). Platelet-rich fibrin scaffolds for cartilage and tendon regenerative medicine: from bench to bedside. Int J Mol Sci.

[21] Qiao J, An N (2017). Effect of concentrated growth factors on function and Wnt3a expression of human periodontal ligament cells in vitro. Platelets.

[22] Inchingolo F, Hazballa D, Inchingolo AD, Malcangi G, Marinelli G, Mancini A (2022). Innovative concepts and recent breakthrough for engineered graft and constructs for bone regeneration: a literature systematic review. Materials.

[23] Bozkurt Doğan Ş, Öngöz Dede F, Ballı U, Atalay EN, Durmuşlar MC (2015). Concentrated growth factor in the treatment of adjacent multiple gingival recessions: a split-mouth randomized clinical trial. J Clin Periodontol.

[24] Hong S, Li L, Cai W, Jiang B (2019). The potential application of concentrated growth factor in regenerative endodontics. Int Endod J.

[25] Li X, Yang H, Zhang Z, Yan Z, Lv H, Zhang Y (2019). Concentrated growth factor exudate enhances the proliferation of human periodontal ligament cells in the presence of TNF-α. Mol Med Rep.

[26] Fang D, Long Z, Hou J (2020). Clinical application of concentrated growth factor fibrin combined with bone repair materials in jaw defects. J Oral Maxillofac Surg.

[27] Fiorentino S, Roffi A, Filardo G, Marcacci M, Kon E (2015). European definitions, current use, and EMA stance of platelet-rich plasma in sports medicine. J Knee Surg.

[28] Del Fabbro M, Bortolin M, Taschieri S (2011). Is autologous platelet concentrate beneficial for post-extraction socket healing? A systematic review. Int J Oral Maxillofac Surg.

[29] Boswell SG, Cole BJ, Sundman EA, Karas V, Fortier LA (2012). Platelet-rich plasma: a milieu of bioactive factors. Arthroscopy.

[30] Chen J, Jiang H (2020). A comprehensive review of concentrated growth factors and their novel applications in facial reconstructive and regenerative medicine. Aesthetic Plast Surg.

[31] Honda H, Tamai N, Naka N, Yoshikawa H, Myoui A (2013). Bone tissue engineering with bone marrow-derived stromal cells integrated with concentrated growth factor in Rattus norvegicus calvaria defect model. J Artif Organs.

[32] Isobe K, Watanebe T, Kawabata H, Kitamura Y, Okudera T, Okudera H (2017). Mechanical and degradation properties of advanced platelet-rich fibrin (A-PRF), concentrated growth factors (CGF), and platelet-poor plasma-derived fibrin (PPTF). Int. J. Implant Dent..

[33] Lei L, Yu Y, Han J, Shi D, Sun W, Zhang D (2020). Quantification of growth factors in advanced platelet-rich fibrin and concentrated growth factors and their clinical efficacy as adjunctive to the GTR procedure in periodontal intrabony defects. J Periodontol.

[34] Yu B, Wang Z (2014). Effect of concentrated growth factors on beagle periodontal ligament stem cells in vitro. Mol Med Rep.

[35] Jin R, Song G, Chai J, Gou X, Yuan G, Chen Z (2018). Effects of concentrated growth factor on proliferation, migration, and differentiation of human dental pulp stem cells in vitro. J Tissue Eng.

[36] Chen X, Chen Y, Hou Y, Song P, Zhou M, Nie M (2019). Modulation of proliferation and differentiation of gingiva-derived mesenchymal stem cells by concentrated growth factors: potential implications in tissue engineering for dental regeneration and repair. Int J Mol Med.

[37] Chen X, Wang J, Yu L, Zhou J, Zheng D, Zhang B (2018). Effect of concentrated growth factor (CGF) on the promotion of osteogenesis in bone marrow stromal cells (BMSC) *in vivo*. Sci Rep.

[38] Takeda Y, Katsutoshi K, Matsuzaka K, Inoue T (2015). The effect of concentrated growth factor on rat bone marrow cells *in vitro* and on calvarial bone healing *in vivo*. Int J Oral Maxillofac Implants.

[39] Yılmaz O, Özmeriç A, Alemdaroğlu KB, Celepli P, Hücümenoğlu S, Şahin Ö (2018). Effects of concentrated growth factors (CGF) on the quality of the induced membrane in Masquelet’s technique – an experimental study in rabbits. Injury.

[40] Borsani E, Buffoli B, Bonazza V, Brunelli G, Monini L, Inchingolo F (2020). *In vitro* effects of concentrated growth factors (CGF) on human SH-SY5Y neuronal cells. Eur Rev Med Pharmacol Sci.

[41] Jun H, Lei D, Qifang Y, Yuan X, Deqin Y (2018). Effects of concentrated growth factors on the angiogenic properties of dental pulp cells and endothelial cells: an *in vitro* study. Braz Oral Res.

[42] Liu Y, Kalén A, Risto O, Wahlström O (2002). Fibroblast proliferation due to exposure to a platelet concentrate *in vitro* is pH dependent. Wound Repair Regen.

[43] Durmuşlar MC, Balli U, Dede FÖ, Misir AF, Bariş E, Kürkçü M (2016). Histological evaluation of the effect of concentrated growth factor on bone healing. J Craniofac Surg.

[44] Park HC, Kim SG, Oh JS, You JS, Kim JS, Lim SC (2016). Early bone formation at a femur defect using CGF and PRF grafts in adult dogs: a comparative study. Implant Dent.

[45] Isler SC, Soysal F, Ceyhanlı T, Bakırarar B, Unsal B (2018). Regenerative surgical treatment of peri-implantitis using either a collagen membrane or concentrated growth factor: a 12-month randomized clinical trial. Clin Implant Dent Relat Res.

[46] Kim TH, Kim SH, Sándor GK, Kim YD (2014). Comparison of platelet-rich plasma (PRP), platelet-rich fibrin (PRF), and concentrated growth factor (CGF) in rabbit-skull defect healing. Arch Oral Biol.

[47] Rochira A, Siculella L, Damiano F, Palermo A, Ferrante F, Carluccio MA (2020). Concentrated growth factors (CGF) induce osteogenic differentiation in human bone marrow stem cells. Biology.

[48] Kizilaslan S, Karabuda ZC, Olgac V (2020). The effect of concentrated growth factor on calvarial bone in diabetic healing. J Craniofac Surg.

[49] Liu C, Xiong H, Chen K, Huang Y, Huang Y, Yin X (2016). Long-term exposure to pro-inflammatory cytokines inhibits the osteogenic/dentinogenic differentiation of stem cells from the apical papilla. Int Endod J.

[50] Zhou C, Zhang X, Xu L, Wu T, Cui L, Xu D (2014). Taurine promotes human mesenchymal stem cells to differentiate into osteoblast through the ERK pathway. Amino Acids.

[51] Onak G, Şen M, Horzum N, Ercan UK, Yaralı ZB, Garipcan B (2018). Aspartic and glutamic acid templated peptides conjugation on plasma modified nanofibers for osteogenic differentiation of human mesenchymal stem cells: a comparative study. Sci Rep.

[52] Barrick B, Campbell EJ, Owen CA (1999). Leukocyte proteinases in wound healing: roles in physiologic and pathologic processes. Wound Repair Regen.

[53] Davis VL, Abukabda AB, Radio NM, Witt-Enderby PA, Clafshenkel WP, Cairone JV (2014). Platelet-rich preparations to improve healing. Part I: workable options for every size practice. J Oral Implantol.

[54] Anitua E, Andia I, Ardanza B, Nurden P, Nurden AT (2004). Autologous platelets as a source of proteins for healing and tissue regeneration. Thromb Haemost.

[55] Pantic I, Paunovic J, Pejic S, Drakulic D, Todorovic A, Stankovic S (2022). Artificial intelligence approaches to the biochemistry of oxidative stress: current state of the art. Chem Biol Interact.

[56] Eming SA, Hammerschmidt M, Krieg T, Roers A (2009). Interrelation of immunity and tissue repair or regeneration. Semin Cell Dev Biol.

